# Statins induce lethal effects in acute myeloblastic lymphoma cells within 72 hours

**DOI:** 10.1080/10428190701760011

**Published:** 2008-01-31

**Authors:** Lillian P. Burke, Cynthia A. Kukoly

**Affiliations:** Department of Internal Medicine, Brody School of Medicine, East Carolina University, Greenville, NC, USA

**Keywords:** Acute myeloid leukemia, statins, HMG CoA reductase inhibitors, drug studies, leukemic cell lines, HL60 cells, U937 cells

## Abstract

HMG-CoA Reductase inhibitors (statins) induce apoptosis in acute myeloid leukemia (AML) cells in vitro; however, the concentrations associated with cell death in AML cells are higher than those clinically tolerated during prolonged therapy. We therefore wished to determine whether short exposures to lovastatin might induce cell death in AML cells at clinically attainable concentrations. The time and concentration dependence of HL60 and U937 cells was determined and showed that cell death was delayed. IC_50_ values and IC_90_ values determined on day 6 suggested that the sensitivity of AML cells to statins may occur at lower concentrations than previously reported. After 72 h, mevalonate did not rescue AML cells from cytotoxic concentrations of statins, suggesting that, although cell death was delayed, lovastatin induced lethal effects within 72 h. In conjunction with previously reported Phase I studies, the data presented here suggest that the high-dose, short course statins may be useful for the treatment of patients with AML.

## Introduction

Statins are useful for the treatment of patients with hypercholesteremia and are among the most widely prescribed drugs. Meta-analyses suggest that statin treatment may decrease the risk of developing certain malignancies although recent studies have provided data conflicting with this hypothesis [1,2]. In vitro, statins induce apoptosis in both primary cells and in tumor cells derived from patients with a variety of malignancies [3]. Sensitivity to statin-induced cyto-toxicity is tumor-line specific. Sensitive cells include cells derived from AML, including chemotherapy-resistant AML [4], as compared with the relative statin resistance seen in cells derived from patients with acute lymphoid leukemia [5,6]. Drug resistant cells may be more sensitive to statins than the non-drug resistant cells, suggesting that statins may be useful for the treatment of drug-resistant malignancies [4,7–9].

Statins induce cell death in malignant cells at concentrations that are significantly higher than those that are effective for the treatment of hypercholesteremia [10]. Cell death occurs via apoptosis and lovastatin concentrations of up to 5 mM and are required to induce apoptosis in AML cell lines and in primary AML cells [5,11,12]. Six of 7 AML cell lines had an IC_50_ of <5.3 mM when exposed to statins for 2 days [5,13]. Primary AML cells also showed a decrease in proliferation and in colony formation when exposed to statins for 2 days, although several of the cultures required lovastatin concentrations of up to 40 mM or more to effect a decrease prolification and or colony formation. In contrast, normal hematopoie-tic cells are tolerant of lovastatin or atorvastatin concentrations of up to 160 mM or more [5,14,15].

These laboratory findings suggest that lovastatin concentrations of approximately 5 mM or more might be required to induce cell death in AML cells. Unfortunately, this lovastatin concentration is at the upper limits of tolerability in human studies, except when the duration of exposure is limited to four days or less [16]. Although several animal species tolerate prolonged duration of statin exposure at concentrations of 2–4 mM [15,17], humans do not tolerate drug concentrations in this range for more than 4–7 days. In a Phase I study of lovastatin, given at up to 25–45 mg/kg/day for 7 days, the *peak* lovastatin concentration in any patient was reported as 3.9 mM [18]. At higher lovastatin concentrations, or with more prolonged duration of therapy, non-hematological toxicities, such as myopathy, hepato-toxicity and renal failure are dose limiting [19]. In contrast, when the lovastatin exposure was limited to a 4 day course of treatment, peak concentrations of up to 12 mM were tolerated without evidence of clinical toxicity [16]. Taken together, these studies suggest that patients might tolerate lovastatin concentrations of up to 12 mM, or possibly more, if the duration of statin exposure is limited to 4 days or less.

However, it is not known whether short courses of high concentrations of statins would be therapeuti-cally efficacious. As a first step in examining these questions, we sought to determine the time and concentration dependence of statin-induced cyto-toxicity for AML cell lines in order to determine the optimal duration of statin exposure for clinical studies in patients with AML. The results reported here support the development of clinical trials of short-course, high dose statins for the treatment of AML.

## Materials and methods

### Cell lines

Cell lines used were obtained from ATCC (Mana-ssas, VA; HL60 and U937 cells). Cells were grown in RPMI 1640 (Gibco, Carlsbad, CA, #11875-093) with 10 mM Hepes and 1 mM sodium pyruvate, supplemented with 10% fetal calf serum (FCS, Hyclone, Logan, UT). All cell lines were maintained at 378C in a humidified atmosphere with 5% CO_2_.

### Reagents

Mevalonate was obtained as mevalonolactone (Sigma, St. Louis, MO M4667). Stock concentrations were made in DMSO and later diluted in media or PBS prior to use in culture. Lovastatin was obtained from CalBiochem, (San Diego, CA), and was converted to the open acid form prior to use as described by Hancock [20]. The resulting lyophilized statins were resuspended in DMSO (Dimethyl sulfoxide, Catalog # D8418, Sigma, St. Louis, MO) and stored in small aliquots at 7 208C until time of use. DMSO at the highest concentration used for the lovastatin treatment served as the vehicle control.

### Cell proliferation

“**MTS assay**”: Cells were seeded in 96 well flat-bottomed plates (100 mL/well, Falcon). For all assays, we determined the number of cells to be plated so that the control cells would be experiencing log phase growth and would be at a concentration so that the assay was in the linear portion of the absorbance curve on the assay (typically 3000 cells/ well). Cell proliferation assays to determine IC_50_ concentrations were measured using the MTS-based assay by Promega (Cell Titer96 Aqueous One Solution, Madison, WI), according to the manufacturer's instructions. All samples were run in triplicate and absorbance recorded at 490 nm with a plate reader after incubation at 378C for 1–3 h. Background absorbance was corrected by subtracting the absorbance values from the media alone (control) wells. This assay is dependent on intracellular metabolism, and absorbance will be decreased if cell proliferation is inhibited, or if the cells are dead or dying. All values were expressed as a percent of the control (untreated) cells unless otherwise specified. For prolonged incubations, drying of the samples was avoided by the addition of sterile, distilled water in the outer wells of microtiter plates.

### Apoptosis detection

At the indicated time, the cells were harvested, stained with the Annexin-V-Fluos (Catalog # 1 828 681, Roche Diagnostics, Indianapolis, IN) and propidium iodide (PI) (Catalog P4170, Sigma, St. Louis, MO) and analyzed by flow cytometry using a Becton Dickenson Immunocytometry Systems (San Jose, CA). CellQuest software (Becton Dickenson) was used for the acquisition and analysis of data. Samples were run in duplicate with 10 000 events counted per sample. Viable cells included cells that were negative for both annexin and PI. Viable cell number was determined by cell counts multiplied by the percent of viable cells.

### Determination of IC_50_ values

The concentrations that decreased cell proliferation by 50% (Inhibitory Concentration 50%, IC_50_) or by 90% (IC90 values) were determined using serial dilutions of lovastatin (typically 500-0.01 mM) and were calculated using the Graph Pad PRISM (Version 3.0; San Diego, CA). To determine rescue of cell proliferation from statins, plated cells were treated with statins alone or in the presence of mevalonate (for concentrations, see figure legends). Plates were assayed on the specified day, with day 0 being the first day of drug exposure.

## Results

IC_50_ values for several AML cell lines are reported to be <5 mM [5]. We first attempted to verify that these concentrations were cytotoxic. After 48 h of exposure to 4 mM of lovastatin, Trypan blue uptake was seen in only 7.6% of HL60 cells (data not shown). Even at a relatively high concentration of lovastatin (50 *mM),* we found that only 10.7% of the cells were Trypan blue positive after 48 h of lovastatin exposure. In contrast, after 6 days of culture in the presence of lovastatin, 50 mM, 82.2% of the HL60 cells were Trypan blue positive. Similar results were seen using U937 cell exposed to 50 mM of lovastatin with 7.1% of U937 cells Trypan blue positive at day 2 and 55.6% of the U937 cells Trypan blue positive at day 6. Since significant cell death was seen late, we examined the time dependence and assay-dependence of statin-induced cytotoxicity.

### Time dependence of statin-induced cytotoxicity

**Figure 1.**
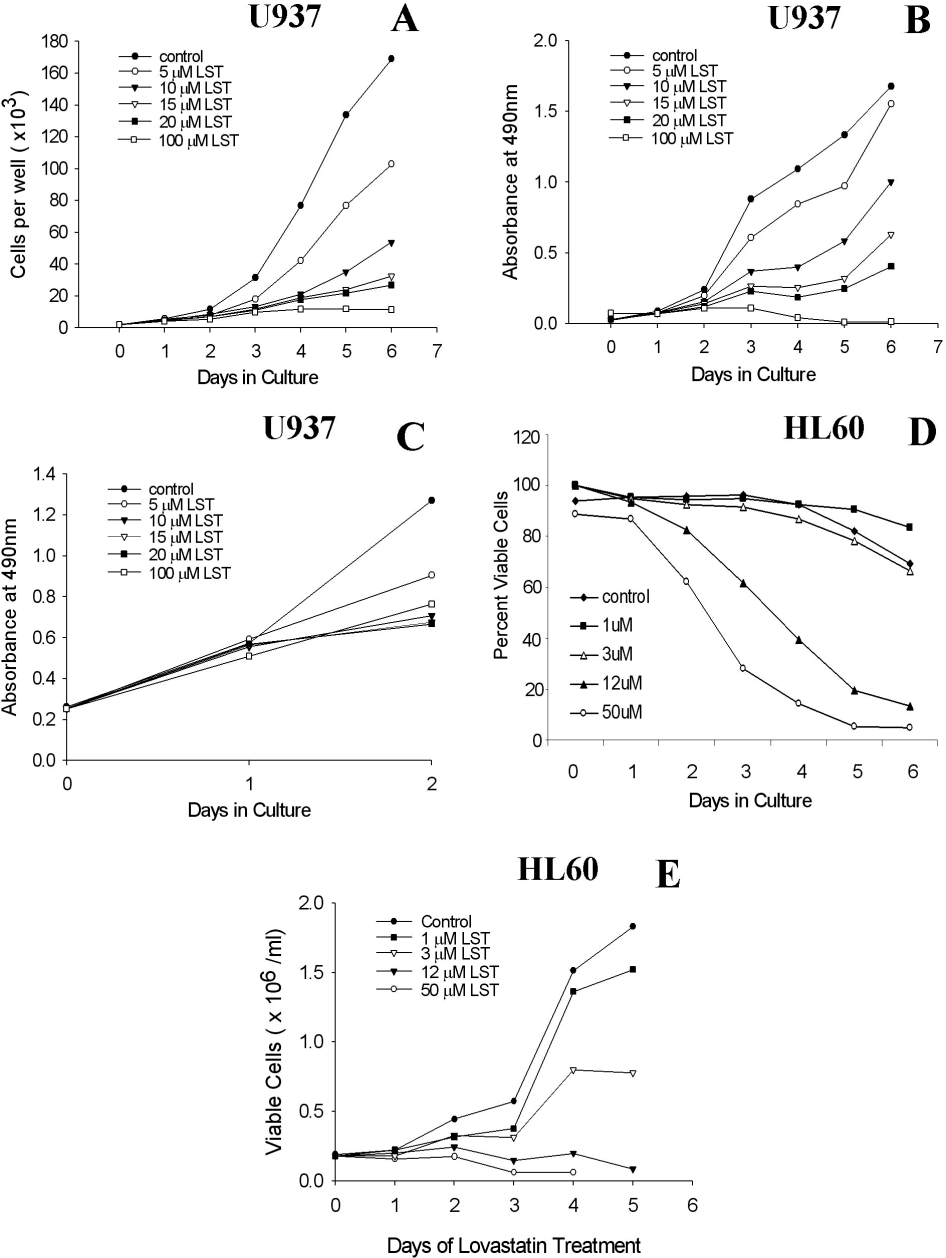
Time course of statin dependent effects on proliferation and AML cell viability. The time dependence of lovastatin (LST) induced effects on proliferation and apoptosis were determined in U937 or HL60 cells. Treated samples were compared with that of untreated cells. (*N*> 3, one example is shown for each assay.) (A) Cells counts by day of culture, at various concentrations. Cell number was determined using a Coulter counter. Shown are total cell counts. At lower concentrations (i.e. 5 15 mM), differences between treated and untreated cells are best seen after at least 4 days of statin exposure. (B) Time course of changes in absorbance (MTS assay). Cell proliferation was measured using an MTS-like colorimetric assay that reflects cellular metabolism. (C) Effects of statins during the first 2 days of culture. Cells were plated at a concentration of 10,000 cells per well in order to look at the effects of lovastatin on proliferation during the first 2 days of drug exposure. These results show that lovastatin did not markedly affect proliferation during the first 2 days of statin exposure, especially at the lower concentrations studied. (D) Percentage of viable cells, by apoptosis, after lovastatin exposure, by day of culture (HL60 cells). Percentage of cells, by day, that were negative for both PI and Annexin. (E) Viable cell number. Using the same data set as shown in 1D, the number of viable cells was determined for each day of treatment by multiplying the number of cells as determined the Coulter counter by the percentage of cells negative for both annexin and PI. In each assay, the cytotoxic effects of concentrations of statins in the clinically relevant range (i.e. <5 —12 mM) are seen only with longer duration of statin exposure.

Proliferation assays are useful for performing replicate assays. It is well-established that statins induce apoptosis in sensitive cells [5,11,21] and apoptosis has been used to detect sensitivity to statins. Therefore, we first determined whether the time course of statin-induced cytotoxicity was the same when assayed by the proliferation assay as compared with cell count and apoptosis assays. U937 cells and HL60 cells were cultured in the presence of serial dilutions of lovastatin. The effects on cell proliferation were determined by both total cell counts [Figure 1(A)] and by the proliferation assay [Figures 1(B) and 1(C)]. Using Annexin V as a marker of apoptosis, we found that full expression of this process is delayed for at least 4–5 days as judged by either the percentage of viable cells [Figure 1(D)] or the total number of viable cells [Figure 1(E)]. In each of these assays, full lovastatin cytotoxic effects were delayed for 4–6 days, especially at lower, more clinically relevant concentrations. Therefore, the delay in evidence of cytotoxicity is not assay dependent when studied at clinically relevant concentrations of lovastatin (i.e. > 12mM).

### Effect of day of assay on determination of IC_50_

**Figure 2.**
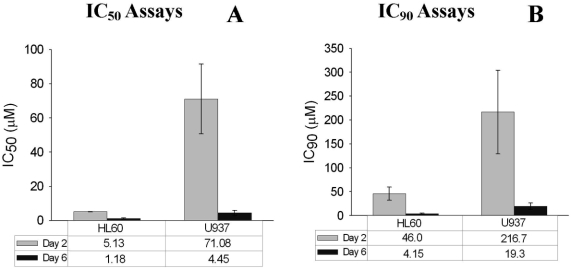
Lovastatin sensitivity, as measured by IC50 and IC90 determinations at days 2 and 6. The values represent the mean + SEM (*N* > 3). (A) IC_50_ values (B) IC_90_ values.

Since maximal cell death was seen after 4–6 days of lovastatin exposure, it was possible that determinations of cytotoxic drug concentrations at day 2 might over-estimate the statin concentrations required to inhibit cell growth and/or to induce cell death. To assess the magnitude of this effect, we compared the IC50 values as determined at day 2, with those determined on day 6 using two cell lines, HL60 cells and U937 cells (Figure 2). When measured by proliferation, the IC_50_ for HL60 was 5.1 mM, very similar to that found by Dimitroulakos et al. who reported that the IC_50_ for HL60 cells was 5.3 mM when measured at day 2 [5]. However, when measured at day 6, the IC50 for HL60 cells was 1.2 mM (a 4-fold difference). The IC50 values for U937 cells showed the same time dependence with apparent IC50 values of 71.1 mM when measured at day 2 and 4.5 mM at day 6 (a 15-fold difference) [Figure 2(A)].

Ideally, a drug that is cytotoxic for AML cells should induce cell death in < 90% of cells. Using the same experimental data as that shown in Figure 2(A), we estimated the IC_90_ values for HL60 cells and U937 cells [Figure 2(B)]. As expected, the IC_90_ values for HL60 and U937 cells were markedly higher when measured at day 2 than at day 6. At day 2, the IC90 for HL60 cells was 46 mM while at day 6, the IC90 was 4.2 mM. The IC90s for U937 at days 2 and 6 were 217 mM and 19.3 mM, respectively [Figure 2(B)].

### Statin exposure for 48–72 h induces cell death in AML cells

Although statin-induced cytotoxicity was maximal at day 6, it was not clear whether this effect was due to prolonged exposure to lovastatin, or due to delayed evidence of cytotoxicity. To explore this question, we used mevalonate to rescue cells from lovastatin. Statins inhibit the enzyme, HMG-CoA reductase, and thereby decrease the production of mevalonate and its downstream metabolites. Mevalonate, when added to statin-containing cultures, inhibits the cytotoxic effect of statins [11,22].

**Figure 3.**
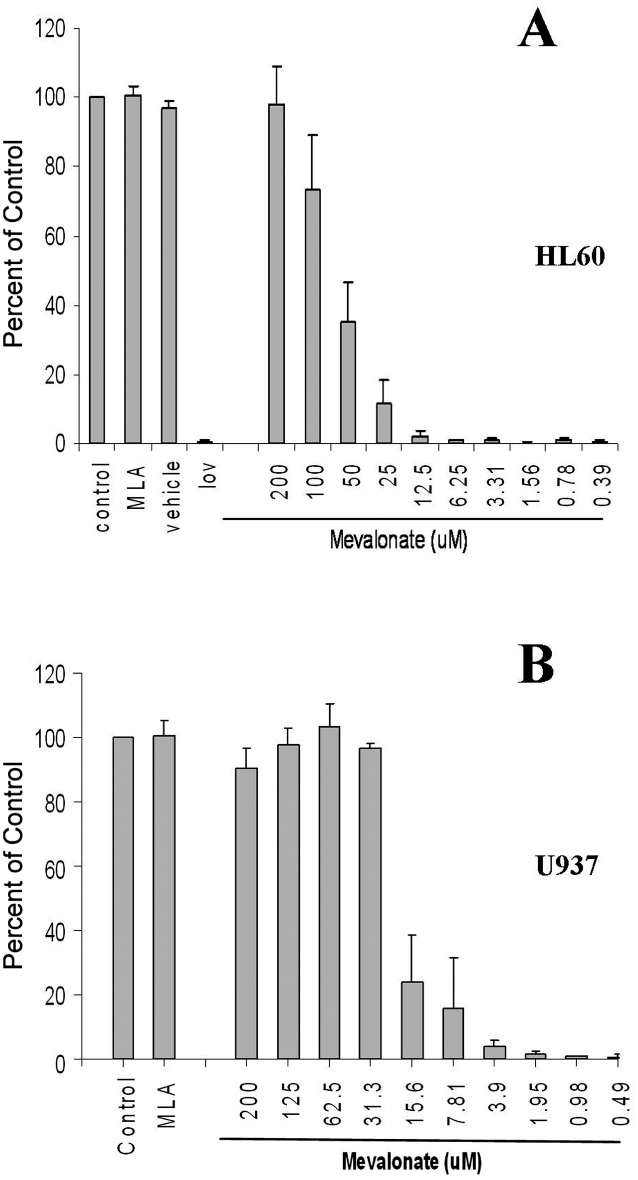
Effects of mevalonate (MLA) on proliferation in the presence or absence of lovastatin (lov). Cells were cultured for 6 days in the presence of lovastatin (50 mM) with or without mevalonate at the stated concentrations. Proliferation was assayed at day 6 by the MTS assay. (*N* ¼ 3 for each cell line) (A) HL60 Cells (B) U937 cells

**Figure 4.**
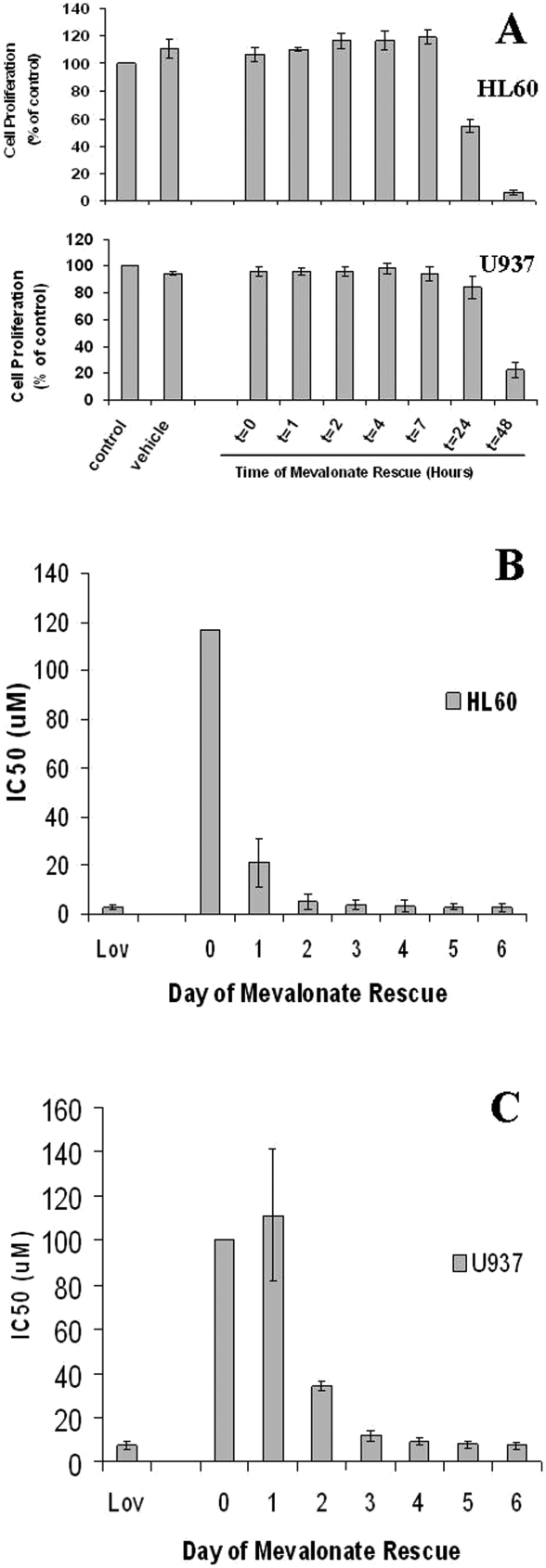
Number of days of statin exposure required for maximal statin-induced cytotoxicity. After various durations of lovastatin exposure, mevalonate was added to the cells to rescue them from the cytotoxic effects of statins in order to determine the minimum duration of statin exposure needed to induce a cytotoxic effect. (A) Duration of lovastatin exposure required at high concentrations (50 mM). HL60 and U937 cells were cultured with 50 M lovastatin for 0, 1, 2, 4, 7, 24 or 48 h prior to the addition of 200 mM Mevalonate. The culture was then incubated for 4 additional days and then assayed using the MTS-based proliferation assay *(N>3* for each cell line). (B and C): IC50 determinations by day of rescue. Mevalonate was added on the indicated day of lovastatin treatment. IC50 values were determined on day 6 (N ¼ 3 for each cell line). (B) HL60 Cells. (C) U937 Cells.

At concentrations of up to 200 mM, mevalonate alone neither increased nor decreased the proliferation of HL60 or U937 cell lines (data not shown). At 200 mM, mevalonate fully rescued both HL60 and U937 cells from the cytotoxic effects of a high concentration of lovastatin (50 mM) (Figure 3). To determine the minimum duration of lovastatin exposure needed to induce statin cytotoxicity, we cultured HL-60 and U937 cells in the presence of lovastatin. After variable durations of lovastatin exposure, mevalonate at 200 mM was added to the cultures in an attempt to rescue the cells (Figure 4). First, we defined the minimum lovastatin exposure time needed to induce lethality by exposing the cells to a high concentration of lovastatin (50 mM) and rescuing the cells with mevalonate (200 mM) after 0, 1, 2, 4, 7, 24 or 48 h of lovastatin exposure [Figure 4(A)]. The effects on cell proliferation were examined at day 6. As expected, mevalonate completely rescued cell proliferation when added to the cultures at time 0. However, after 48 h of exposure to this high concentration of lovastatin, mevalonate was not able to rescue either HL60 or U937 cells from statin-induced cytotoxicity.

We next examined the time dependence of lower concentrations of lovastatin by determining the effect of the duration of unopposed lovastatin (before mevalonate rescue) on the IC_50_ values. When mevalonate was added after 2 days of statin exposure, the IC50 for HL60 cells was almost the same as that seen after 6 days of unrescued exposure to statins [Figure 4(B)]. U937 cells required 72 h of statin exposure before rescue in order to have an IC50 that was similar to that seen after 6 days of statin exposure without mevalonate rescue [Figure 4(C)].

### AML cells do not recover from lovastatin exposure after 3 days

**Figure 5.**
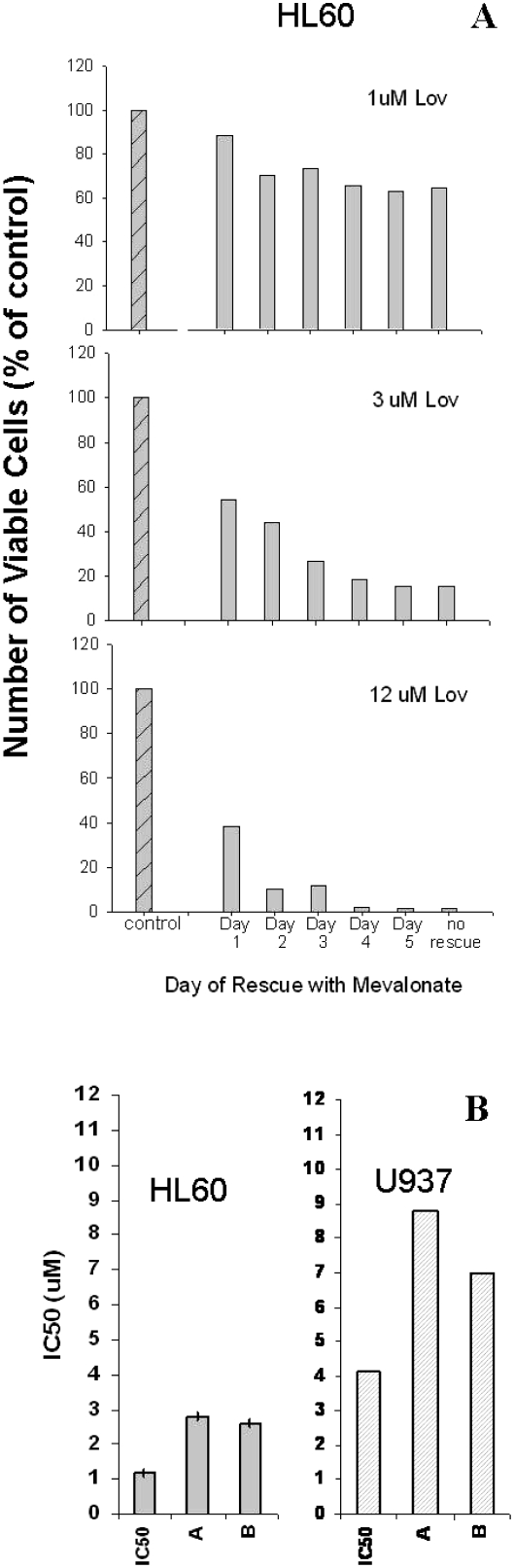
Effect of lovastatin pre-treatment on viability at clinically attainable concentrations. (A) Viability as assessed by Trypan blue staining: HL60 cells were treated with 1, 3 or 12 mM lovastatin on Day 0. Mevalonate (200 mM) was added to the cells on day 1, 2, 3, 4 or 5. The effects on viability determined on day 6 as measured by cell counts and Trypan blue uptake. *N*¼ 2; one example is shown. (B) Ability to recover from lovastatin after 72 h of exposure. HL60 or U937 cells were exposed to lovastatin for 72 h and then rescued with mevalonate (200 mM). They were allowed to proliferate for an additional 6 days. The IC_50_ was determined after a total of 3 days of lovastatin exposure and 6 days of mevalonate-rescued growth, i.e. on day 9. Two separate assays were performed and the results for each are as shown (*N*¼ 2 for each cell line, with the individual samples indicated by “A” or “B”). For comparison purposes, the IC50 values as determined previously are plotted (same data as Figure 2, day 6).

To determine whether lovastatin induced cell death at low concentrations or merely inhibited proliferation, we examined the viability of statin-exposed cells using Trypan blue staining of HL60 cells at day 6, when rescued after various durations of statin exposure. At 12 mM of lovastatin, most of the HL60 cells were non-viable after 48 h of statin exposure as the number of viable cells that were Trypan blue negative (i.e. viable) in the treated cultures was only 10.4% that of the control cultures [Figure 5(A)]. We also looked at the ability of the AML cells to recover from lovastatin by incubating the cells for 72 h in the presence of serial dilutions of lovastatin. The cells were then rescued with mevalonate and allowed to recover for 6 days. Despite the prolonged recovery period, the IC50 values for both U937 cells and HL60 cells were similar to the IC50 values of the unrescued cells [Figure 5(B)].

## Discussion

High dose statins may be useful for the treatment of AML, but the optimal drug concentration and duration of therapy required to induce cell death in AML cells is not known. Dimitroulakos et al. determined the IC50 values of several AML primary cells and cell lines and showed that six of seven of these AML cell lines had IC50 values between 1.0 and 5.3 mM when assayed after 2 days of lovastatin exposure [3]. Our initial studies showed that most cells appeared to be viable after 2 days of statin exposure as determined by Trypan blue staining. This finding was consistent with the original data published in other studies [5,13,23]. Statins are known to decrease cell proliferation; however, at concentrations higher than 5 mM, statins may induce apoptosis, resulting in cell death [24]. These initial studies suggested that single-agent statins might not induce cell death at clinically tolerable concentrations.

As reviewed above, Phase I studies suggest that the upper concentration of lovastatin that is clinically tolerable is approximately 4 mM for 7 days [18] or 12 mM for 4 days [16] but these concentrations are not tolerated when the duration of exposure is prolonged [19]. An alternative way to improve the therapeutic efficacy of high dose statins for the treatment of AML might be to increase the drug concentration while decreasing the duration of statin exposure. However, the ability of statins to induce cell death in AML cells after a short exposure has not been clear. In these studies, we demonstrate that lovastatin induces cell death in sensitive AML lines within 72 h of exposure to cytotoxic concentrations.

Our initial studies showed that evidence of statin-induced effects on cell proliferation and viability were delayed. Maximal cytotoxic effects were not seen for at least 4 to 6 days, depending on the particular assay (Figure 1). Delayed effects were seen in assays of proliferation and apoptosis, as well as traditional studies based on cell counts and Trypan blue staining. Delays were also seen at high concentrations of statins. The delay in evidence of statin-induced cytotoxicity affected the determination of apparent cytotoxic drug concentrations. The IC50 values as determined at day 2 were 4-fold (HL60 cells) or 15-fold (U937 cells) higher than those determined at day 6 [Figure 2(A)]. The estimates of IC90 values showed similar discrepancies, with a 10-fold increase in apparent required concentrations of statins when the assays were performed at day 2 as compared with day 6 [Figure 2(B)]. Thus, measurement of the IC_50_ at day 2 over estimates the actual IC_50_ and IC_90_ concentrations by ½ log or more. Most significantly, the IC_50_ and IC90 values determined at day 6 predict that lovastatin will induce cell death in AML cells at clinically tolerable concentrations in contrast to the less encouraging findings when these values are determined at day 2, especially for the less sensitive U937 cells [Figure 2(A)].

Although cytotoxicity was evident at clinically tolerable concentrations at day 6, it was not clear whether this increase in sensitivity at day 6 was due to longer duration of drug exposure or due to delayed evidence of statin effects. To distinguish the effects of longer statin exposure from the effects of delayed cell death, we used mevalonate to rescue cells from statins [4]. Mevalonate alone, as a single agent, neither increased nor decreased AML cell proliferation (Figure 3). At a high concentration of lovastatin (50 mM), only 48 h of unopposed drug exposure was required to block mevalonate rescue of both HL60 and U937 cells [Figure 4(A)]. However, 50 mM lovastatin is not likely to be clinically tolerable. For this reason, we next explored the relationships among duration of lovastatin exposure before mevalonate rescue and the IC50 values. These studies demonstrated that when mevalonate rescue was delayed for 48 h [HL60 cells, Figure 4(B)] or 72 h [U937 cells, Figure 4(C)], the IC_50_ values were essentially the same as the IC50 values of unrescued cells exposed to lovastatin for 6 days [Figures 4(B) and 4(C)]. These results were not assay dependent as similar results were seen using Trypan blue staining of HL60 cells exposed to clinically relevant concentrations of lovastatin [Figure 5(A)]. Therefore, although full evidence of statin-induced cell death is delayed for 5–6 days, statins induce irreversible lethal effects in AML cells within 48–72 h.

Since lovastatin inhibits cell proliferation, it was also possible that these cells were not dead but simply had stopped proliferating and did not have time to recover. To test this possibility, we exposed the AML cells to lovastatin for 72 h, then rescued the cells with mevalonate and allowed them to proliferate for a full 6 days after mevalonate rescue. Even after 6 days of mevalonate rescue, neither HL60 cells nor U937 cells were able to resume proliferation (Figure 5).

Taken together, these results suggest that AML cells with sensitivity similar to HL60 and U937 cells might undergo cell death at lovastatin concentrations of 12 mM or less, and that this effect is irreversible within 48–72 h of exposure to cytotoxic concentrations of this drug. The clinical importance of these findings is that they imply that at least some cells will have IC_50_ values, or even IC_90_ values, at concentrations that might be clinically tolerable for 2–3 days (i.e. <12 mM). Furthermore, statins at clinically tolerable concentrations do not merely inhibit cell proliferation; rather, they induce cell death. One limitation of our studies is that these have been performed only in AML cell lines. Studies in primary cells are presently ongoing; however, previous reports indicate that the statin sensitivity of primary cells corresponds well to that of AML cell lines [5].

The time dependence of statin-induced cytotoxi-city has been previously noted as an incidental finding in AML cells [5]. In myeloma cells, simvastatin and flustatin increase the sensitivity to cytotoxic agents in a time-dependent manner [25]. Although statins affect activation of the MAPK signaling pathway within 6 h, induction of caspase 3 is not seen for 48 h [26].

In vitro, a few days of lovastatin exposure synergistically increases the sensitivity of malignant cells to chemotherapy. This effect may be more marked in drug-resistant cell lines [4,27]. Clinically, statins seem to be well-tolerated in combination with cytotoxic agents as the toxicities are non-overlapping. A Phase I trial of pravastatin, in combination with idarubicin and cytarabine produced encouraging responses in patients with high-risk leukemia [28]. Van der Spek et al. treated patients with simvastatin 15 mg/kg/day for 7 days before chemotherapy with VAD or CHOP regimens for myeloma or lymphoma, respectively, with an overall response rate of 30% in a heavily pre-treated population and minimal evident toxicity [25]. Forty-eight to 72 h of lovastatin or simvastatin exposure sensitized AML cells to cytarabine [29,30, for example]. Therefore, these preliminary clinical and laboratory studies suggest that a short course of high dose statins can be tolerated in combination with cytotoxic agents.

In summary, we found that the full cytotoxic effect of lovastatin in AML cells was not seen for at least 4–6 days after exposure to lovastatin. Despite the delayed evidence of cell death, lovastatin induces irreversible lethality after 48–72 h of drug exposure. These irreversible toxic effects occur at clinically tolerable concentrations of <12 mM for both HL60 and U937 cells. In conjunction with the results of Phase I studies, these results suggest that high-dose, short course statins might induce cell death in AML cells at plasma concentrations that are clinically tolerable if high dose statin exposure is limited to <4 days. These studies also provide theoretical support for the development of additional clinical studies to examine the tolerability and efficacy of high-dose, short-course statins for the treatment of patients with AML.
