# Erratum

**DOI:** 10.1080/10428190802076630

**Published:** 2008-04-08

The publishers would like to apologise for an error that occurred in the title of the following article, printed in Leukemia & Lymphoma, February 2008; 49(2): 322–330.

Statins induce lethal effects in acute myeloblastic lymphoma cells within 72 hours

LILLIAN P. BURKE & CYNTHIA A. KUKOLY

It should have read:

Statins induce lethal effects in acute myeloblastic leukemia cells within 72 hours

LILLIAN P. BURKE & CYNTHIA A. KUKOLY

